# Partial Response to Nivolumab and Ipilimumab in a Patient With Malignant Pleural Mesothelioma and Pre‐Existing Myasthenia Gravis Without Severe Flares or Immune‐Related Adverse Events: A Case Report

**DOI:** 10.1002/rcr2.70297

**Published:** 2025-08-19

**Authors:** Mariko Higa, Tomoya Kuda, Yuichiro Ohya, Hidenori Kawasaki

**Affiliations:** ^1^ Division of Pulmonary Medicine NHO Okinawa Hospital Ginowan Okinawa Japan; ^2^ Division of Pulmonary Medicine Okinawa Prefectural Chubu Hospital Uruma Okinawa Japan; ^3^ Division of Neurology NHO Okinawa Hospital Ginowan Okinawa Japan; ^4^ Department of Surgery NHO Okinawa Hospital Ginowan Okinawa Japan

**Keywords:** disease flares, immune‐related adverse events, ipilimumab, nivolumab, pre‐existing myasthenia gravis

## Abstract

Detailed clinical data on the combination immune checkpoint inhibitor (ICI) therapy in patients with myasthenia gravis (MG) remain limited. We report a case of malignant pleural mesothelioma with previously undiagnosed ocular MG. Owing to hepatic dysfunction, reduced doses of nivolumab and ipilimumab were administered before confirmation of anti‐acetylcholine receptor (AChR) antibody positivity. MG was diagnosed based on subtle ocular symptoms and serological tests. Prophylactic intravenous immunoglobulin and an acetylcholinesterase inhibitor were administered; combination therapy was discontinued owing to the risk of MG flare, but a partial tumour response was achieved. With disease progression, nivolumab monotherapy was reintroduced, and early steroid pulse therapy was administered owing to elevated creatine kinase, again inducing a partial response. Serological screening for anti‐AChR antibodies may help prevent severe MG flares and immune‐related adverse events. With caution, dose‐reduced and limited‐exposure ICI combination therapy may be feasible in selected patients with MG under appropriate prophylactic management.

## Graphical abstract

A patient with malignant pleural mesothelioma and pre‐existing myasthenia gravis (MG) was treated with reduced‐dose nivolumab plus ipilimumab, followed by reintroduction of nivolumab. Both treatment phases resulted in a partial response without severe MG exacerbations or immune‐related adverse events. This rare case emphasises the potential feasibility of immune checkpoint inhibitor (ICI) therapy in patients with MG.

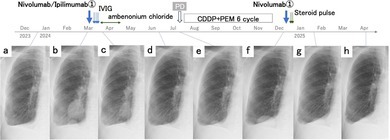

## 1 Introduction

Combination immune checkpoint inhibitor (ICI) therapy with nivolumab, targeting programmed cell death protein 1 (PD‐1), and ipilimumab, targeting cytotoxic T‐lymphocyte–associated antigen 4 (CTLA‐4), improves survival in malignant pleural mesothelioma (MPM). It is now recommended as a first‐line treatment [1]. Myasthenia gravis (MG)—an autoimmune disease affecting the neuromuscular junction—is generally excluded from combination ICI therapy because of the high risk of severe immune‐related adverse events (irAEs) [2].

## 2 Case Report

**FIGURE 1:**
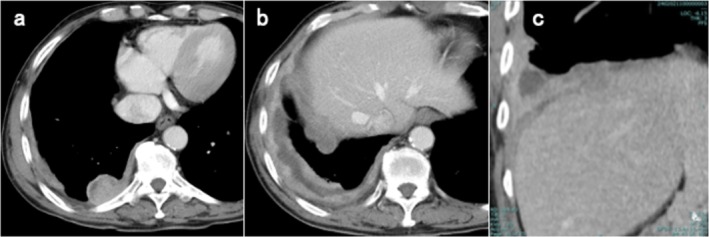
Contrast‐enhanced computed tomography images obtained 30 days before nivolumab plus ipilimumab administration. (a) Shows a pleural‐based mass in the dorsal segment of the right lower lobe; (b) shows pleural thickening, effusion, and multiple masses at the right lung base. (c) A coronal reconstruction of (b), showing blurring of the medial right hemidiaphragm, suggestive of liver invasion.

**FIGURE 2:**
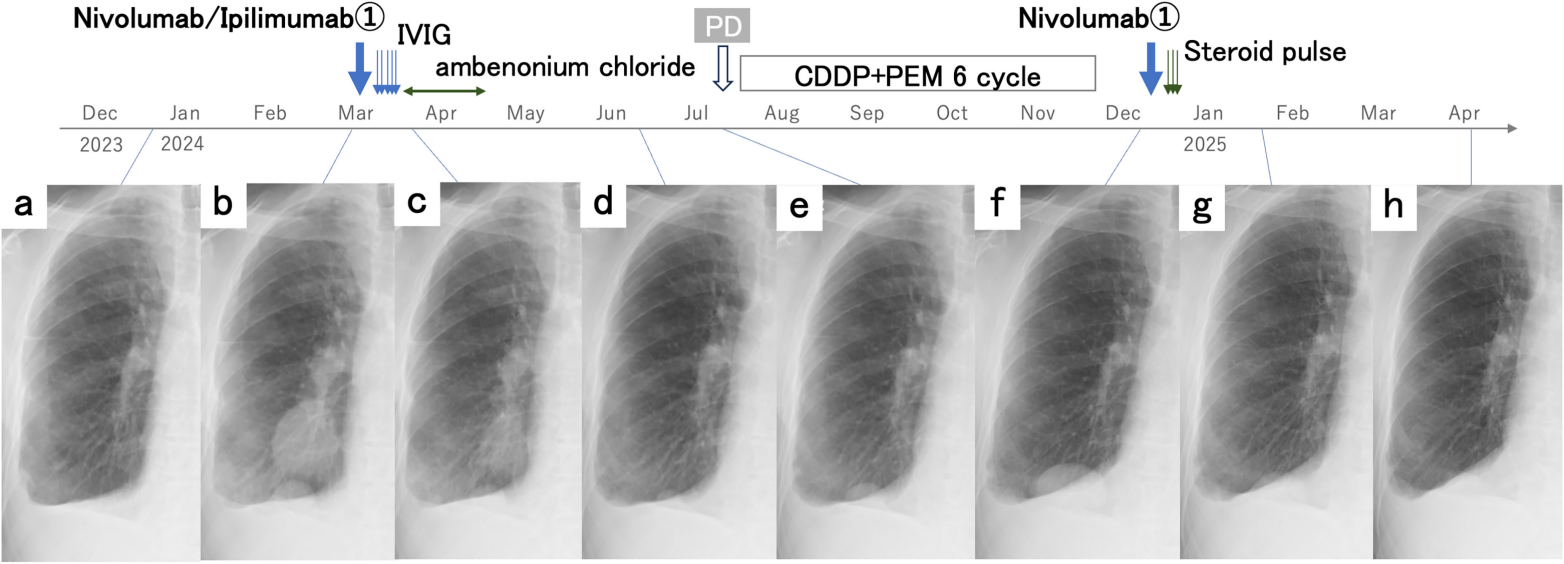
Treatment timeline and serial chest radiographs. (a) Seventy‐seven days before nivolumab plus ipilimumab administration. (b) Three days before administration, showing rapid tumour growth over approximately 2 months. (c) Fourteen days post‐administration, showing tumour shrinkage. During this period, prophylactic treatment for myasthenia gravis flare (intravenous immunoglobulin [IVIG] and oral ambenonium chloride) was administered. (d) On Day 101 post‐administration, a partial response (PR) was maintained; however, by Day 130, enlargement of the basal lung mass was observed, and progressive disease (PD) was diagnosed (e). Cisplatin (CDDP) and pemetrexed (PEM) were subsequently administered for six cycles, resulting in stable disease as the best‐observed response; however, the mass gradually increased. (f) One day before nivolumab monotherapy. Steroid pulse therapy was initiated in response to elevated CK levels on Day 7 after administration. (g) On Day 45, post‐nivolumab monotherapy, shrinkage of the basal lung mass was observed, and PR was sustained through Day 114.

A man in his 70s with stage IIIB biphasic MPM and liver invasion (Figure 1) was referred to our department. He had no history of autoimmune disease (AID). Serological screening for AID was performed before initiating ICI therapy. Owing to rapid disease progression (Figure 2a,b) and the absence of MG symptoms, treatment commenced before anti‐acetylcholine receptor (AChR) antibody results became available. The standard regimen (nivolumab 360 mg every 3 weeks and ipilimumab 1 mg/kg every 6 weeks) was reduced to 240 mg and 0.5 mg/kg, respectively, owing to hepatic dysfunction.

Anti‐AChR antibody positivity was confirmed the day after infusion. Neurological examination revealed diplopia on lateral gaze and left‐sided ptosis, which temporarily improved with the ice pack test. Detailed history analysis revealed that symptoms had been present for 4–5 years. Consequently, the patient was diagnosed with pre‐existing ocular MG (MG Foundation of America class I). Although the symptoms were sufficiently minor that the patient had not noticed them, intravenous immunoglobulin (IVIG) at a total dose of 2 g/kg was administered on Days 8–12 to prevent deterioration. Oral ambenonium chloride, an acetylcholinesterase inhibitor, was initiated on Day 15.

Frequent ventricular and atrial premature contractions were noted between Days 18 and 22, raising concern for myocarditis. However, electrocardiography was normal; creatine kinase (CK) levels ranged from 59 to 73 U/L; troponin T was negative; and echocardiography revealed a preserved left ventricular ejection fraction (64%) without regional wall motion abnormalities. Arrhythmias resolved after Day 23, and myocarditis was ruled out. ICI therapy was discontinued due to the risk of an MG flare. MG symptoms remained stable without exacerbation, and ambenonium chloride was discontinued on Day 44.

Chest radiography on Day 14 showed shrinkage of right middle‐to‐lower lung masses (Figure 2c), with a partial response maintained until Day 130 (Figure 2d,e). Subsequent regrowth of the right lower lung mass was classified as a progressive disease (Figure 2e). The patient underwent six cycles of cisplatin and pemetrexed, achieving stable disease as the best response.

With further tumour progression (Figure 2f), the patient and his family opted for nivolumab monotherapy, fully understanding the potential risk of MG exacerbation. As MG was in remission, no prophylactic or baseline MG treatment was administered. The patient remained asymptomatic after 240 mg nivolumab, although CK levels increased to 861 U/L on Day 7. No muscular or chest symptoms suggestive of myositis or myocarditis were observed. To prevent MG flare and ICI‐related myositis, steroid pulse therapy with methylprednisolone (1 g/day) was administered for 3 days beginning on Day 7. CK levels normalised, and the patient remained asymptomatic, but the therapy was discontinued owing to flare risk. Partial response was observed again and has been maintained to date (Figure 2g,h).

## 3 Discussion

Although PD‐L1 expression has been explored as a potential predictive biomarker in MPM, its clinical utility remains controversial. In the CheckMate 743 trial, first‐line combination therapy with nivolumab and ipilimumab significantly prolonged overall survival compared to platinum‐based chemotherapy, regardless of PD‐L1 expression status. Therefore, dual ICI therapy is now recommended for unresectable MPM, and routine PD‐L1 testing is not mandatory for treatment selection [1].

This case suggests that combination ICI therapy may be effective and tolerable in selected patients with pre‐existing MG when carefully managed. ICI monotherapy in patients with MG shows favourable response rates (71.4% partial and 7.1% complete remission) [3]. In patients with pre‐existing AIDs, combination therapy is associated with a trend toward longer progression‐free survival compared to monotherapy [4].

However, MG exacerbation occurs in 70.8% of patients with MG treated with ICI monotherapy [3]. In the management of MG relapse, ICI therapy was discontinued in 88.2% of patients, all of whom were treated with corticosteroids. Among them, 64.7% additionally received IVIG or plasmapheresis. Complete resolution of relapse was achieved in 29.4% of patients, while 47% showed incomplete resolution and 23.5% experienced a fatal outcome [3]. Combination therapy may further increase the risk of severe irAEs such as myositis and myocarditis. A report indicates that 6 of 7 patients with MG treated with PD‐1/CTLA‐4 inhibitors developed these complications [5], a pattern sometimes referred to as the ‘triple‐M complication’ (myasthenia, myositis, and myocarditis), which is known for its high mortality and rapid progression. Unlike nivolumab, ipilimumab shows a dose/exposure‐dependent relationship with adverse events, appearing to be markedly enhanced when used in combination with nivolumab [6]. Furthermore, as most severe MG flare cases are reported during the first or second cycle of ICI therapy [5], a limited strategy, particularly involving dose reduction of ipilimumab and restricting its use to a single administration, may represent a reasonable approach. The rechallenge of ICI therapy in MG remains controversial. Among 110 patients who developed de novo MG after ICI, only 9 in remission resumed treatment. All continued prophylactic therapy with corticosteroids, pyridostigmine, and IVIG. MG recurrence was observed in only one case, and irAEs occurred in two cases. Partial or complete tumour responses were achieved in seven patients [5]. Although one case involved nivolumab re‐administration following combination therapy, detailed clinical data are unavailable [5]. In carefully selected patients with well‐controlled MG and no viable alternative therapies, ICI rechallenge may be cautiously considered. Current guidelines do not recommend screening for anti‐AChR antibodies before initiating ICI therapy [2]. Mild symptoms may go unrecognised by patients. However, in this case, screening enabled timely MG detection. Prophylactic use of IVIG and an acetylcholinesterase inhibitor following diagnosis, combined with early recognition of potential worsening based on elevated CK levels and prompt initiation of steroid pulse therapy before overt exacerbation, likely contributed to stable MG disease control.

In conclusion, although caution is warranted, combination ICI therapy may be feasible in selected patients with MG when administered with dose reductions, limited exposure, and appropriate prophylactic measures.

## Author Contributions

Mariko Higa: writing. All authors: editing and final review.

## Consent

The authors declare that written informed consent was obtained for the publication of this manuscript and accompanying images using the form provided by the Journal.

## Conflicts of Interest

The authors declare no conflicts of interest.

## Data Availability

The data that support the findings of this study are available on request from the corresponding author. The data are not publicly available due to privacy or ethical restrictions.
